# Improvement of Targeted Fungi Secondary Metabolite Production Using a Systematic Experimental Design and Chemometrics Analysis

**DOI:** 10.3390/mps6050077

**Published:** 2023-08-29

**Authors:** Lamonielli F. Michaliski, Laura P. Ióca, Leandro S. Oliveira, Camila M. Crnkovic, Mirelle Takaki, Vitor F. Freire, Roberto G. S. Berlinck

**Affiliations:** 1 Instituto de Química de São Carlos, Universidade de São Paulo, CP 780, São Carlos CEP 13560-970, SP, Brazil; lamonielli.michaliski@usp.br (L.F.M.); laura.ioca@alumni.usp.br (L.P.I.); leandroquimica17@gmail.com (L.S.O.); mirelle_takaki@outlook.com (M.T.); vitorffreire@gmail.com (V.F.F.); 2 Departamento de Tecnologia Bioquímico-Farmacêutica, Faculdade de Ciências Farmacêuticas, Universidade de São Paulo, São Paulo CEP 05508-000, SP, Brazil; camilavic@usp.br

**Keywords:** secondary metabolites, natural products, fungi, microorganism, chemometrics, metabolomics, fermentation yield, fractional factorial design

## Abstract

Fungi are well-known producers of chemically diverse and biologically active secondary metabolites. However, their production yields through fermentation may hamper structural analysis and biological activity downstream investigations. Herein, a systematic experimental design that varies multiple cultivation parameters, followed by chemometrics analysis on HPLC-UV-MS or UHPLC-HRMS/MS data, is presented to enhance the production yield of fungal natural products. The overall procedure typically requires 3–4 months of work when first developed, and up to 3 months as a routine procedure.

## 1. Introduction

Secondary metabolites, also known as natural products, encompass a diverse group of compounds, numbering over 340,000 in the *Dictionary of Natural Products* (as of May 2023). Such metabolites are divided into seven established categories, i.e., polyketides, peptides, terpenoids, alkaloids, purine and pyrimidine-derived, phenylpropanoids, and hybrid metabolites (biosynthesized by mixed metabolic pathways) [1]. Secondary metabolites are produced by all living organisms, from bacteria to humans [1,2]. Several secondary metabolites display potent biological activities which are important for human [3,4], animal [5], and plant [6,7] health. Secondary metabolites also present ecological roles, such as chemical defenses [8,9,10] and semiochemicals [11]. Consequentially, natural products are of much interest in several branches of academic science and industrial applications [12].

Among the biological sources of secondary metabolites, fungi harbor an enzymatic machinery capable of biosynthesizing all classes of natural products [13]. Nevertheless, the fungal metabolic expression is frequently only a fraction of its genomic capabilities, varying upon cultivation conditions [14,15]. Therefore, approaches to activate biosynthetic gene clusters and/or to increase the production yield of minor, structurally unique bioactive fungal metabolites are of interest for the microbial natural product community [16,17]. For example, the OSMAC strategy (“one strain many compounds”) has been shown to activate silent gene clusters in microbial culture by changing the nutrient availability during the fermentation process [15,18]. On the other hand, strategies using statistical methods have been successfully applied to overcome the low yields of microbial fermentation of already known useful metabolites [19] and in the discovery of new ones [20,21,22,23].

Surprisingly, to the best of our knowledge, previously to our investigations there are no reports on improvements in the production of secondary metabolite mixtures in a systematic way, by varying multiple culture conditions at once. In the past decade, we have been applying a combination of statistical approaches to optimize fungal culture conditions using a fractional factorial experimental design (FFED) along with chemometrics analysis. This strategy enabled us to improve the production of several fungal secondary metabolites. For example, from an optimized culture condition for *Penicillium citrinum* F53, the new alkaloids citrinalins A and B were identified, isolated, and their biosynthesis investigated [24,25]; this strategy also improved the production of meleagrine and oxaline to investigate their biosynthesis [24]; it also allowed the identification of sulfur-bearing macrolactones by the marine-derived *Penicillium* sp. DRF2 [26], and the yield improvement of a unique polyketide produced by the *Roussoella* sp. DLM33 fungal strain [27] and of the first nitrogen-bearing representative of phomactins, which are inhibitors of the platelet aggregation factor [28,29].

Herein, details of the protocol to apply this systematic FFED and chemometrics methodology are presented based on our previous investigations [24,25,26,27,28,29]. The overall method is described, aiming to provide information on how to improve the fungal production of secondary metabolites in liquid cultures after a group of metabolites or a specific compound have been previously selected after dereplication. The step-by-step procedure is described, including the statistical analysis to transform data into information that allows yield optimization for secondary metabolite production.

## 2. Experimental Design

In order to develop a reliable method to enhance the production yields and the chemical diversity of fungal metabolites produced in liquid culture, the following protocol includes seven steps: 1. reactivate a fungal strain from a preserved stock; 2. prepare a spore suspension; 3. acquire and evaluate preliminary data of a suitable chemical profile; 4. select responses factors and variables; 5. perform a fractional factorial experimental design; 6. perform data acquisition and chemometrics analysis to determine the enhanced culture parameters; and 7. validate the growth conditions, as predicted.

Yet, a few questions should be asked before planning the FFED: First, what is the expected output? If (a) the output is to enhance the chemical diversity, the response factors should be the overall mass yield of a specific fraction containing the desired metabolites, the number of chromatographic peaks corresponding to metabolites and chromatographic peak areas of metabolites under a standardized HPLC-UV-MS analysis. If (b) the output is to increase the production of one or a few specific compounds, the response factors should be the mass yield of fraction(s) containing those metabolite(s), and the chromatographic area corresponding to the target peak(s) visualized after HPLC-UV-MS or UHPLC-HRMS/MS analysis. Second, what are the fungal, or microbial, culturing parameters which could influence this output? In our strategy, media composition and culture conditions were selected as FFED variables, such as salts and nutrient concentration, along with time, mode (shaking or still), and temperature of growth, since we found that these parameters most affected the secondary metabolite production by the fungal strains we have previously investigated [24,26,27]. Other variables can be included, e.g., pH or the addition of elicitors or epigenetic molecules, as reported elsewhere [15,24,30].

If the aim is to enhance the production of a target compound, it is presumed that the chemistry of the fungal strain has been previously investigated. However, if nothing is known about the metabolites produced by the strain, we recommend applying the OSMAC strategy to find an appropriate preliminary culture condition [15,29] prior to the FFED approach, aiming to select the initial medium composition and growth parameters to be improved.

**Figure 1:**
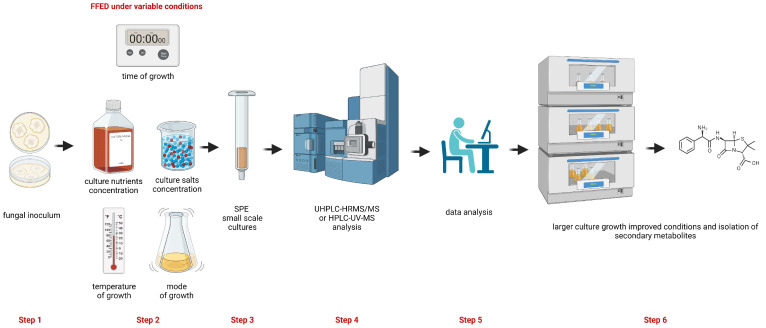
Diagram of the steps included in the protocol to improve the diversification and/or the production yield of fungal secondary metabolites. Step 1: recovery of the fungal culture; step 2: performing cultures of the selected fungal strain under different variables (time of growth, temperature of growth, culture nutrients concentration, culture salts concentration, and mode of growth (shaking or still)); step 3: extraction and SPE clean-up of extracts from the fungal cultures; step 4: analysis of SPE fractions by UHPLC-HRMS/MS or by HPLC-UV-MS; step 5: chemometrics analysis; step 6: selection of the improved conditions of fungal cultures growth for a large scale culture towards the isolation of secondary metabolites. This figure was prepared using a free version of BioRender^®^.

This protocol includes the following steps (Figure 1):

1. Recovery of the preserved fungal strains and preparation of fungal spore suspension.
2. Small-scale cultivation of fungal strain(s) under selected variable conditions of an FFED (second discussion point above).
3. Growth media extraction and clean-up using solid-phase extraction (SPE) of extracts obtained from culture media of FFED experiments.
4. Analysis of target fractions obtained after SPE using HPLC-UV-MS or UHPLC-HRMS/MS procedures.
5. Chemometrics analysis of the acquired data.
6. Establishing improved conditions determined by FFED and chemometrics analysis for isolation of target secondary metabolites.

If the fungal strain has not been previously chemically screened or investigated, the overall procedure is completed within 4 months considering a maximum time for the fungal growth of 40 days. Since some fungal strains can grow and produce metabolites in shorter periods, e.g., 7 days, the overall time of the optimization process can be considerably reduced. The disadvantage observed in this strategy is the time invested. It can vary according to the fungi’s growth periods, number of parameters to be modified, and laboratory infrastructure availability. The method is of interest to microbiology specialists, natural product and pharmacognosy researchers, as well as professionals devoted to work on drug discovery, food chemistry, agriculture biocontrol, microbial metabolism, cosmetics, fermentation products, and biotechnology.

### 2.1. Materials

1. Malt extract (Acumedia^®^, San Bernardino, CA, USA, cat. no. 7341);
2. Papaic digest of soybean meal 2 (Peptone S2, Acumedia^®^, San Bernardino, CA, USA, cat. no. 9213);
3. Potato Dextrose Broth (Acumedia^®^, cat. no. 7585);
4. Agar powder, bacteriological grade (Himedia^®^, Mumbai, India, cat. no. RM026);
5. Boric acid (HBO_3_, Synth, cat. no. A1025);
6. Calcium chloride dihydrate (CaCl_2_·H_2_O, Synth, cat. no. C1041);
7. Magnesium chloride hexahydrate (MgCl_2_·6H_2_O, Synth, cat. no. C1051);
8. Potassium bromide (KBr, Synth, cat. no. B1023);
9. Potassium chloride (KCl, Synth, cat. no. 1058);
10. Sodium chloride (NaCl, Synth, cat. no. C1060);
11. Sodium phosphate dibasic (NaH_2_PO_4_, Vetec, cat. no. V000317) (**CAUTION!** Acute toxicity, eye, and skin irritation);
12. Sodium sulfate (Na_2_SO_4_, Synth, cat. no. S1071);
13. Sodium bicarbonate (NaHCO_3_, Synth, cat. no. B1011);
14. Strontium chloride hexahydrate (SrCl_2_·6H_2_O, Synth, cat. no. C1060);
15. Celite 545 (Synth, cat. no. C1016);
16. Distilled water (reverse osmosis, Tecnal, TE 4007-10);
17. Ultrapure water (0.22 µm, Milli-Q^®^—Millipak^®^, cat. no. MPGL040011);
18. Methanol, UV-IR-HPLC grade, P.A.—A.C.S. (MeOH, Panreac, cat. no. 361091) (**CAUTION!** Highly flammable, toxic and health hazard);
19. Acetonitrile, UV-IR-HPLC grade, P.A.—A.C.S. (MeCN, Panreac, cat. no. 361881) (**CAUTION!** Highly flammable and toxic);
20. Ethyl acetate, P.A.—A.C.S. (EtOAc, Synth, cat. no. A1010) (**CAUTION!** Flammable);
21. Dichloromethane, P.A.—A.C.S. (CH_2_Cl_2_, Synth, cat. no. D1003) (**CAUTION!** Toxic);
22. Ethanol, P.A.—A.C.S. (EtOH, Synth, cat. no. A1084) (**CAUTION!** Highly flammable);
23. Formic acid 98%, P.A.—A.C.S. (Panreac, Darmstadt, Germany, cat. no. 131030) (**CAUTION!** Corrosive and flammable);
24. Tween 80—U.S.P. (Synth, cat. no. T1029).

### 2.2. Equipment

- Eppendorf tubes;
- Microscope (Leica DM500, Wetzlar, Germany);
- Neubauer improved counting chamber, 0.0025 mm^2^ (Precicolor HBG, Munich, Germany, cat. no. 9030-01);
- pH meter (ION pH B500, New York, NY, USA);
- Scale (Sartorius, Shinagawa City, Tokyo);
- Sterile plastic Petri dishes, 90 × 15 mm (Cralplast, Cotia, Spain, cat. no. 18248A);
- Parafilm^®^ “M” (Sigma-Aldrich^®^, Saint Louis, MO, USA, cat. no. P7668-1EA);
- Pasteur pipette, 230 mm (Uniglas, Houston, TX, USA, cat. no. 180.230);
- Schott^®^ flask (250 and 500 mL, Uniglass, Houston, TX, USA);
- Vertical autoclave AV (Phoenix Luferco, Araraquara, Brazil, Model AV-75, serie no. 19233);
- Laminar flow hood (Pachane, Maharashtra, India, Pa420);
- Infra-red sterilizer (Biotec^®^, Pathum Thani, Thailand, Model BT 1201, series no. 130537);
- Incubator for BOD TE-3911 (Tecnal, Medellín, Colombia);
- Incubator Shaker Series (Innova^®^ 44, Duluth, GA, USA);
- Threaded culture tubes, 20 × 125 mm (Pyrex^®^, Rosemont, IL, USA, cat. no. 9824-20);
- Test tubes, 20 × 150 mm (Pyrex^®^, Rosemont, IL, USA, cat. no. 99445-20);
- Centrifuge tube, 50 mL, 30 × 115 mm, EO sterile (Corning^®^, New York, NY, USA);
- Pipette, 100 and 1000 mL (Eppendorf Research plus);
- Pipette tip, 1–10 µL, 10–200 µL, 100–1000 µL and 1–10 mL (Axygen Scientific Inc., Union City, CA, USA);
- Sterile syringe, 5 mL (Plastipack™, Ann Arbor, MI, USA);
- Vacuum Manifold Pump (KNF Laboport^®^, cat. no. ASME B40.1);
- Vacuum concentrator, SpeedVac^®^ Plus (ThermoSavant, Cham, Switzerland, cat. no. SC210A);
- Refrigerated Vapor Trap (ThermoSavant, cat. No. RVT4104);
- Deep vacuum oil pump, ValuPump (ThermoSavant, cat. no. VPL80);
- Rotary evaporator (Sibata, Japan);
- Pump LR37697 (SA^®^);
- Paper filter, 80 g/m^2^, diameter 11 cm (Unifil, Beirut, Lebanon);
- Ultrasonic cleaner, 40 kHz (Unique, model USC-2800);
- Fiberglass;
- Centrifuge (Eppendorf, model 5810R);
- PTFE membrane filter, diameter 47 mm, pore size 0.45 µm (Sigma-Aldrich^®^, cat. no. P0825);
- C_18_ reversed-phase cartridges, 2 g (12 mL), 5 g (20 mL) and 10 g (60 mL), 55 µm—70 Å (Stracta—Phenomenex^®^);
- Reversed-phase Waters C18 X-terra column (50 mm or 250 mm length, 5 µm).

## 3. Procedure (Indicating the Approximate Time to Perform Each Step)

### 3.1. Recovery of a Fungal Strain (1–2 Weeks)

1. Take 50 µL of a Castellani solution (Box A1) of the fungal strain under investigation, or a piece of the fungus mycelium, and place it over a Petri dish previously prepared with a nutrient rich solid medium (e.g., M3 or PDB media). **CRITICAL STEP!** To avoid any contamination, this step must be carried out in a laminar air flow cabinet.
2. Seal the Petri dish with Parafilm^®^, label and store it at room temperature or in an incubator at a suitable temperature (often at 25 °C). Usually, the growth of fungal cultures in Petri dishes takes 1–2 weeks. The fungal growth on the Petri dish must be periodically verified. **CRITICAL STEP!** If a contamination or unusual morphology of the fungal culture in the Petri dish is noticed, step 1 must be repeated.

### 3.2. Prepare a Spore Suspension (1–10 Days)

3. Transfer the fungus from the Petri dish culture(s) to slant culture tubes using a sterile loop. At least 10 tubes are necessary in this step. Fungi usually take 3–10 days to produce spores. Slant culture tubes should be stored at the appropriate temperature for growth. It is recommended to prepare the slant tube with 3–4 mL of PDB medium in a 10 mL test tube. **CRITICAL STEP!** To prevent any contamination, all steps must be carried out under a laminar air flow cabinet. The loop should be sterilized with an infrared sterilizer. Optionally, a gamma-irradiated sterile disposable inoculating loop can be used. Some fungal strains do not produce spores, if this is the case, skip steps 3 to 9 and see **TROUBLESHOOTING**, Table 1.
4. After the fungal strain growth period, add 5 mL of a 0.5% Tween 80 solution into each slant culture tube. Scrape the mycelium surfaces off with a sterile loop (10–15 cm long).
5. Filter the suspension obtained through sterile fiberglass into a sterile Erlenmeyer flask containing small glass pearls. Carefully shake the Erlenmeyer flask to release the spores from the sporangium. **CRITICAL STEP!** All material must be previously sterilized using an autoclave. A sterile syringe can be used to hold the fiberglass. Pressure should not be applied to speed up the filtration step.
6. Split the filtered suspension equally in two centrifuge tubes. Seal the tubes with Parafilm^®^.
7. Centrifuge the tubes at 4000 rpm at room temperature for 15 min.
8. Discard the supernatant. Resuspend the pellets (containing spores) in 15 mL of sterile distilled water. Repeat step 7. Repeat step 8 three times.
9. Combine the pellets in one spore suspension. Count the spore suspension using a Neubauer improved counting chamber (see Box A2).

### 3.3. Evaluation of Secondary Metabolite Production (2–3 Weeks)

10. If the fungal strain has not yet been chemically investigated, a small-scale cultivation of the fungal strain must be performed to evaluate the chemical profile produced in culture media, e.g., we highly suggest applying the OSMAC methodology for a first assessment of a fungal metabolic outcome under different nutrient conditions. Herein, M3 medium is used as an example. Inoculate the spore suspension at a final concentration of approximately 10^5^ spores.mL^−1^ into 250 mL Schott^®^ flasks containing 50 mL of M3 medium. This experiment must be performed in triplicate. Alternatively, if the fungal strain does not sporulate, 3 mycelium disks from a solid culture are inoculated into the liquid medium (see **TROUBLESHOOTING**, Table 1). **CRITICAL STEP!** To prevent any contamination, this step must be carried out under a laminar air flow cabinet using sterile pipette tips. Schott^®^ flasks must not be closed tightly to allow aeration of the culture(s).
11. Harvest the culture media after 3 weeks of growth. Shorter or longer periods of growth can be applied, depending on the fungal strain (**TROUBLESHOOTING**, Table 1). After growth, different media extraction procedures can be used. The following procedure has been adopted by us over several years, to generate an extract from the (a) whole growth media or; (b) from the EtOAc-soluble organic fraction of the growth media.
  - Filter the fungal culture through a celite pad over a filter paper in a Büchner funnel placed on the top of a Kitassato flask connected to a vacuum system. Concentrate the filtered culture medium in a rotatory evaporator or in a high-vacuum apparatus (Speedvac) until dry. Optionally, the filtrate can be freeze-dried. The dried filtered media is subsequently subjected to an SPE using C_18_-derivatized silica gel cartridges. **CAUTION!** Cartridges with the correct amount of the stationary phase must be used based on the amount of dried filtrate obtained: up to 100 mg sample (1 g cartridge), from 101 to 200 mg (2 g cartridge), from 201 to 500 mg (5 g cartridge), from 501 to 1000 mg (10 g cartridge). Cartridges should be pre-conditioned with MeOH, 1:1 MeOH/H_2_O, and 100% H_2_O prior to use. Dilute the sample in 1–2 mL of H_2_O. If the dried filtrate is not soluble in 100% H_2_O, see **TROUBLESHOOTING**, Table 1. After cartridge conditioning, apply the sample on the top of the cartridge stationary phase. Elute the column with the following MeOH/H_2_O gradient (**CAUTION!** The volume of each liquid phase will depend on the cartridge size, here we considered a 2 g cartridge): 100% H_2_O (discarded, contains media hydrosoluble components, salts and/or fungal primary metabolites); 25:75 MeOH/H_2_O (10 mL, F1), 50:50 MeOH/H_2_O (10 mL, F2), 75:25 MeOH/H_2_O (10 mL, F3) and 100% MeOH (10 mL, F4). Evaporate all fractions to dryness under vacuum, then transfer to pre-weighed labeled vials and weigh each fraction.
  - To each Schott^®^ flask with 50 mL of media, add 50 mL of EtOAc. Sonicate the mixture at 40 kHz for 30 min to 1 h. Let it sit overnight. Filter the mixture culture medium + EtOAc through a celite pad, as described in (a). Separate the EtOAc organic phase by liquid–liquid partition and evaporate it to dryness. Resuspend the dried EtOAc extract into 100 mL of 95:5 MeOH/H_2_O and degrease it by partitioning (3×) with the same amount of hexane. Dry the methanolic extract and transfer it to a pre-weighed labeled vial and weigh it.
12. Prepare 1 mg.mL^−1^ solutions of each of the SPE fraction or methanolic extract obtained in step 11 in MeOH. Analyze all samples using HPLC-UV-MS using a Waters C_18_ X-terra column (250 × 4.6 mm, 5 µm), or similar, with a linear gradient of 10–100% of 1:1 MeOH/MeCN in H_2_O with 0.1% formic acid for 30 min, with a 1 mL.min^−1^ flow rate.
13. The extraction method, either (a) or (b), can be selected based on the results observed after analysis by HPLC-UV-MS.

### 3.4. Fractional Factorial Experimental Design (1–3 Months)

14. A 2^5−1^ fractional factorial experimental design (FFED) starts by defining five factors to be selected in a series of the fungus growth experiments, aiming to improve the production of secondary metabolites. When we applied FFED for our particular purposes, the selected variables were total concentration of inorganic salts in the ASW preparation (see Reagents Setup), designed as [salts]; time of growth (t), in number of days; temperature of growth (T), in °C; growth under shaking (in rpm) or still mode; total concentration of nutrients of M3 growth medium (see Reagents Setup), designed as [nutrient]. Variables were tested in two levels, +1 (high) and −1 (low), as defined in Table A1. The number of growth experiments (n = 16) is defined as 2^n−1^, where n = number of variables (n = 5). Additional or different variables can also be used, e.g., growing the strain under light or in the dark, changing specific media components, by adding (or not) additives that may stimulate the expression of biosynthetic gene clusters (e.g., epigenetic modifiers), among others (**TROUBLESHOOTING**, Table 1). In every case, the maximum (+) and minimum (−) levels of each variable must be defined.
15. The conditions for each growth experiment are defined in Table A2, according to the variables selected and their corresponding levels (+ or −). The table columns are filled by alternating each level in column 1 ([salts]), by alternating each two levels in column 2 (t), each four levels in column 3 (T), and each eight levels in column 4 (mode), in agreement with standardized chemometrics procedures [31]. Column 5 ([nutrient]) should be filled with maxima (+) and minima (−) levels using the expression: (level column 1) × (level column 2) × (level column 3) × (level column 4) for each line. This approach will result in the main effects being confounded with quaternary effects, that should be of negligible importance. Table A3 describes the conditions for each growth experiment (GE) that we have performed.
16. A GE is composed of a 250 mL Schott^®^ flask with 50 mL of the liquid culture medium, as defined in Table A3. Each GE growth condition is also defined in Table A3. Inoculate each GE with the fungus spore solution to a final concentration of 10^5^ spores.mL^−1^. **CRITICAL STEP!** To prevent any contamination, this step must be carried out under a laminar air flow cabinet with sterile pipette tips. Schott^®^ flasks must not be closed tightly to allow air exchange. For growth using shaking mode, bind the flask and the lid with adhesive tape to avoid accidental stop air exchange. **CAUTION!** If the fungal strain does not sporulate, inoculate three mycelium disks taken from a culture Petri dish in each Schott flask, using a standard time of growth. In this case, it is recommended to build an accurate calendar to not mix up the inoculum dates.
17. At the end of the growth period for each growth experiment, culture media is extracted following the method selected in step 13. Fractions should be evaporated to dryness, weighed, and stored in a refrigerator at 4 °C until further analysis.
18. Prepare each fraction at the concentration of 1 mg.mL^−1^ (in MeOH). HPLC-UV-MS analyses are performed using a Waters C_18_ X-terra column (50 × 4.6 mm, 5 µm) and a linear gradient of elution from 10 to 100% MeOH/MeCN 1:1 in H_2_O during 12 min. (**TROUBLESHOOTING**, Table 1).
19. For each HPLC-UV-MS analysis, the area under the chromatographic peaks of interest is calculated and the number of chromatographic peaks is counted. These data, along with the weight of each fraction obtained at step 17, are used as the *responses of each growth experiment*. Write down each response for each fraction of each triplicate experiment. **CRITICAL STEP!** If the aim is to improve the production of a single metabolite, the response of the FFED will be the area under the chromatographic peak corresponding to the single compound.

### 3.5. Chemometrics Analysis (1–2 Days)

20. The *desirability function of a multicriteria methodology* converts the three response values into a single unidimensional value (Box A3), named as the *individual desirability* (di). The *global desirability* (D) is the geometric average value of the three individual desirabilities for each GE. D is used to calculate the *first-* and *second-order effects*. A first-order effect measures the influence of one variable on the growth condition, while a second-order effect measures the influence of the interaction between two variables on it.
21. The FFED variables should be numbered as follows: [salts] = 1; t = 2; T = 3; mode = 4; [nutrient] = 5. First-order effects are calculated by multiplying the variable levels (+1 or −1) in each growth experiment by the global desirability (see an example in Table S1).
22. Sum positive (Σy^+^) and negative values (Σy^−^) obtained in each column resulting from step 21 (1 × D, 2 × D, 3 × D, 4 × D, and 5 × D). Divide the sum (Σy^+^ + Σy^−^) by 8 (number of experiments in each level, considering number of variables n = 5, see an example in Table S1). This value corresponds to the first-order effect of that specific variable to enhance the metabolic production for each growth condition.
23. For second-order effects, multiply the level value (+1 or −1) of two variables by the global desirability values (D) for each growth experiment (1 × 2 × D, 1 × 3 × D, 1 × 4 × D, 1 × 5 × D, 2 × 3 × D, 2 × 4 × D, …, 4 × 5 × D; see an example in Table S2). Sum the values obtained in each column (Σy^+^ + Σy^−^) and divide by 8 (Σ/8). This value corresponds to the second-order effect exerted by the interaction of two defined variables to enhance the growth condition.
24. Transform each effect (first- and second-order) into a percentage (Pi). See Box A4 for the complete chemometric procedure.
25. To establish an improved growth condition, the highest percentage first-order effect should be identified. This is the main variable, or the variable that most influences the fungal growth output (production of metabolites). Then, identify this variable level: if (Σ/8) < 0, then the variable at the low level should be used (reference value: −1); if (Σ/8) > 0, then the variable at the high level should be used (reference value: +1).
26. To determine the levels of the other variables for the enhanced growth condition, use the second-order effects between the main variable (reference value) and the other ones as follows. This will depend on the reference value:
  - If reference value is +1, and:
    - If second-order effect value is <0, this variable must be used in the low level (−1).
    - If second-order effect value is > 0, this variable must be used in the high level (+1).
  - If reference value is −1, and:
    - If second-order effect value is <0, this variable must be used in the high level (+1).
    - If second-order effect value is >0, the variable must be used in the low level (−1).
  **CRITICAL STEP!** To avoid any mistake, always check the percentages of the first- and second-order effects. For example, if the reference value of the first-order effect of a variable disagrees with the reference value of the second-order effect between this variable and the main/reference one, use the percentage to make the correct decision. In other words, use the reference number which has the highest percentage. Furthermore, the chemical profile under a HPLC-UV-MS analysis of the specific growth condition must be checked to make sure that the output is as predicted.

27. If the growth condition obtained in step 26 was not included among the 16 growth experiments of the FFED selected experiments (Table A3), this new growth experiment must be performed and numbered as GE #17. The additional experiment should be performed as described in steps 16–18 to experimentally confirm the expected output(s) established by the chemometrics analysis.

### 3.6. Troubleshooting

Troubleshooting advice can be found in Table 1, below.

**Table 1:** Potential issues that can be observed for different steps in the procedures.

Step	Problem	Possible Reason	Solution
3	The fungus does not sporulate.	-	Use 1–3 mycelium disks of a solid culture. Standardize the solid media, period, and temperature of growth.
10	Low-complexity chemical profile observed in the HPLC-UV-MS analysis.	The volume of culture medium is not enough to detect the compounds, due to low yields.	Increase the amount of culture medium produced for each growth experiment. Volumes such as 100 mL, 250 mL, and 500 mL should be tested.
11	Expected chemical profile was not observed.	-	Different extraction procedures must be tested before starting this protocol.
11a	Dried filtrate is not soluble in H_2_O.	-	Solubilize the sample in MeOH and adsorb it in celite (1:1 *m*/*m* is recommended). Subject the celite + sample to careful evaporation (with a gentle flow of nitrogen gas, or in a Speedvac apparatus) until dryness. After drying, place the celite + sample at the top of the cartridge using a spatula.
14	Different number of variables.	-	FFED levels table using different number of variables (n) can be used. Column levels must be alternated accordingly to 2^x−1^, with x = column number up to x = n − 1. Last column level (x = n) is the product of preceding columns.
18	It is difficult to measure the chromatographic area and count the peaks.	Not enough resolution to separate the chromatographic peaks.	Test a different elution gradient, and/or different HPLC column.

## 4. Results

This protocol can be adapted to any microbial culture, such as fungal, bacterial, or cyanobacterial strains. Considering the protocol’s simplicity, robustness, reproducibility, and versatility, it is useful for multiple use purposes related to microbial natural product production.

**Figure 2:**
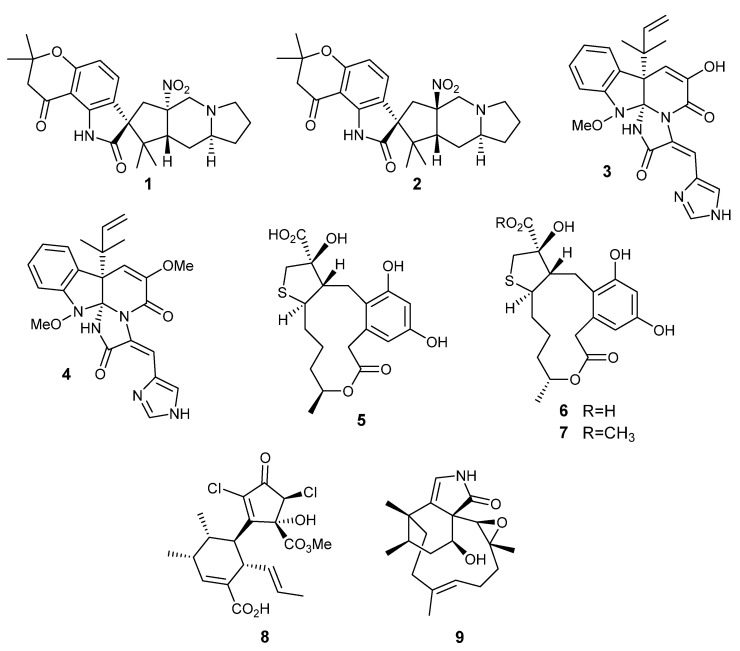
Secondary metabolites isolated and identified after improving fungal culture parameters for *Penicillium citrinum* P53 (**1**,**2**), *P. oxalicum* P30 (**3**,**4**), *Penicillium* sp. DRF2 (**5**–**7**), *Roussoella* sp. DLM33 (**8**), and *Biatriospora* sp. CBMAI 1333 (**9**): citrinalin A (**1**), citrinalin B (**2**), meleagrine (**3**), oxaline (**4**), cyclothiocurvularin A (**5**), cyclothiocurvularin B (**6**), cyclothiocurvularin B methyl ester (**7**), roussoellatide (**8**), and phomactinine (**9**) [24,25,26,27,28,29].

The protocol described above was first applied to improve the growth conditions of *Penicillium citrinum* F53, aiming to diversify and enhance its metabolite production [24]. The outcomes enabled the discovery of citrinalin A (**1**) and B (**2**) [24] and enabled the subsequent investigation of citrinalin’s biosynthesis [25]. Additionally, the production of the alkaloids meleagrine (**3**) and oxaline (**4**) by *P. oxalicum* P30 could be improved [24] and critical steps of their biosynthesis could be investigated, leading to the discovery of a nitrone synthase enzyme [32]. This protocol was subsequently used to improve the metabolic production by *Penicillium* sp. DRF2, leading to the isolation of sulfur-bearing curvularin derivatives (e.g., **5**–**7**) [26]. This protocol was also applied to increase the production of roussoellatide (**8**) by the fungus *Roussoella* sp. DLM33, also enabling its biosynthesis to be investigated [27]. The increase in the production of **8** enabled its isolation in sufficient amount to acquire an INADEQUATE spectrum, an NMR pulse sequence that allows the detection of direct ^13^C-^13^C couplings, very rarely used for natural product identification due to the limited amounts of the compounds that are normally obtained [33]. More recently, the same method was used to increase the yield and to diversify the production of phomactins by the fungus *Biatriospora* sp. CBMAI 1333, enabling the discovery of the first nitrogen-bearing phomactin, phomactinine (**9**) [29] (Figure 2).

### 4.1. Improvement of Citrinalin Production by Penicillium citrinum P53 and of Oxaline and Meleagrin Prodution by Penicillium oxalicum P30

The alkaloids citrinalins A (**1**) and B (**2**), oxaline (**3**), and meleagrine (**4**) were the first metabolites to have their production yields investigated and improved, using the first FFED and chemometrics analysis procedure we developed [24]. Citrinalins belong to a rare group of metabolites, presenting a nitro group connected to an sp^3^ carbon, since most nitro-bearing natural products have the nitro group attached to an aromatic moiety [34]. After application of our first method [24], the biosynthesis of citrinalins could be investigated [28]. Oxaline (**3**) and meleagrine (**4**) also belong to a very rare class of natural products, presenting an sp^3^ carbon connected to three nitrogens. Application of the procedure we developed in its first stage [24] enabled us to discover a unique nitronase enzyme [32].

### 4.2. Improvement of the Production of Curvularin-Related Polyketides by Penicillium sp. DRF2

By performing an FFED and chemometrics analysis to improve the culture conditions of the strain *Penicillium* sp. DRF2, it was possible to increase the production and to isolate minor secondary metabolites belonging to the class of the curvularin macrocyclic lactones condensed with a mercaptolactate in a very unusual tetrahydrothiophene ring moiety (**5**–**7**) [26]. In this case, the production of the mycotoxin citrinin was suppressed by changing the cultivation temperature from 30 °C to 15 °C (compare Figures S1 and S2). The growth in still mode allowed the production of a higher number and higher yields of novel natural products **5**–**7** (compare Figures S2 and S3 or S4 and S5).

### 4.3. Improvement in the Production of Roussoellatide by Roussoella sp. DLM33

Some modifications were performed in this procedure to adapt this protocol to improve the production of roussoellatide (**8**) by *Roussoella* sp. DLM33. The changes are specified in Table A4 [27]. Using the improved culture conditions determined by the FFED for *Roussoella* sp. DLM33, the production yield of compound **8** was doubled when compared with its production under the initial culture growth conditions.

### 4.4. Diversification of Phomactin Terpenoid Production by Biatriospora sp. CBMAI 1333

Additional modifications were made to this protocol to adapt it to improve the chemical diversification and production yields of the phomactin family of terpenoids by the fungal strain *Biatriospora* sp. CBMAI 1333. The changes are specified in Table A5 [29]. The improved culture conditions determined by the FFED for *Biatriospora* sp. CBMAI 1333 led to diversification of phomactin production and detection of potentially new analogs, directing us to the isolation of the first nitrogen-bearing phomactine, phomactinine (**9**) [29].

## 5. Protocol Limitations and Further Applications

Previous methods for the improvement of fungal secondary metabolite production focused almost exclusively on single compounds [35,36]. An earlier, and rare, example of the use of statistical analysis to improve the production of secondary metabolite mixtures by fungi was aimed at the improved production of ergot alkaloids [37]. For single compounds, the use of chemometrics and experimental design approaches proved very successful for the improved production of the statins monacolin, lovastatin, and compactin [38,39,40], as well as of the potently cytotoxic agent squalestatin S1 [41]. However, to the best of our knowledge, our method herein described is the very first applied for the *discovery* of *novel* and bioactive fungal secondary metabolites by using chemometrics and experimental design.

Although we found no drawback in our protocol, it should be noted that each step needs to be carefully designed, performed, and assessed by analyzing the experimental data, to avoid misinterpretations and erroneous conclusions. Because of this, the time for its application is usually between 3 and 4 months. Thus, the fungal strain to be investigated needs to be previously selected by biological and/or chemical screening by using one of various dereplication methods [42]. A careful selection of a fungal strain to be investigated using our method avoids the redundant isolation of known compounds. It can be extremely useful for the enhanced production of bioactive compounds and secondary metabolism diversification, as has been recently demonstrated by us in the diversification of the PAF-inhibitory agents phomactins, that led to the discovery of the first nitrogenated phomactin congener, phomactinine (**9**) [29].

We envision that the use of automation and artificial intelligence tools can potentially accelerate the application of the method herein described, with significant outcomes for the discovery of new fungal metabolites with potential medical applications.

## 6. Conclusions

The application of a simple and inexpensive method based on chemometrics and experimental design led to the discovery of a series of new metabolites produced in fungal cultures. The method proved to be straightforward and of significant impact for the discovery of the new metabolites, e.g., **1**–**9**, produced by different fungal cultures. The method can be easily extended towards the discovery of metabolites produced by different microbial strains, such as bacteria, actinobacteria, myxobacteria and cyanobacteria, well-known producers of biologically active secondary metabolites useful for several applications in human health, in the food industry, and as agrochemicals.

## 7. Reagents Setup

**Artificial sea water (ASW):** This solution is used to prepare liquid culture media to perform the growth of marine-derived microorganisms instead of using natural sea water. When working with non-marine-derived microorganisms, the use of distilled water is preferable. The composition of ASW solution is: 1.36 g of CaCl_2_·H_2_O, 9.68 g of MgCl_2_·6H_2_O, 0.61 g of KCl, 30.0 g of NaCl, 140.0 mg of NaH_2_PO_4_, 3.47 g of Na_2_SO_4_, 170.0 mg of NaHCO_3_, 100.0 mg of KBr, 40.0 mg of SrCl_2_·6H_2_O, and 30.0 mg of HBO_3_ for 1 L of distilled water.

**MF medium:** Composition: 20 g.L^−1^ of glucose, 10 g.L^−1^ of soluble starch, 20 g.L^−1^ of soytone, 5 g.L^−1^ of peptone, 5 g.L^−1^ of yeast extract, 300 mg.L^−1^ of meat extract, in ASW. The initial pH is 8.0. For solid medium, 15.0 g.L^−1^ of agar is added to this recipe.

**Malt 3% (M3) medium:** Composition: 30.0 g of malt extract, 3.0 g of peptone for 1 L of ASW. The initial pH is 8.0. For solid medium, 15.0 g.L^−1^ of agar is added to this recipe.

**Potato dextrose broth (PDB) medium:** Composition: 24.0 g of potato dextrose broth for 1 L of ASW. The initial pH is 8.0. For solid medium, 15.0 g.L^−1^ of agar is added to this recipe.

**CAUTION!** All culture media must be autoclaved before use. Schott^®^ flasks must not be closed tightly during autoclaving. The pressure of the autoclave must be zero atm. before it can be opened to prevent hot steam from emerging. Heat protective gloves should be worn.

**Solution of Tween 80:** Composition: 0.5% of Tween 80 in distilled water.

**CAUTION!** This solution must be autoclaved before use. Schott^®^ flasks must not be closed tightly during autoclaving. The pressure of the autoclave must be zero atm. before it can be opened to prevent hot steam from emerging. Heat protective gloves should be worn.

**Solvents for the extraction of the secondary metabolites produced in culture medium:** Many solvents are employed for extraction procedures, including distilled water, acetonitrile (MeCN, highly flammable and toxic), dichloromethane (CH_2_Cl_2_, harmful), ethyl acetate (EtOAc, highly flammable and irritant), methanol (MeOH, highly flammable and toxic), and formic acid (highly flammable, toxic, serious eye damage, skin corrosion and corrosive to metals). These solvents are all analytical grade.

**CAUTION!** All solvents must be handled carefully. Wear protective clothing, safety glasses, and gloves. Solvents should be handled under a fume hood and stored in a solvent cabinet.

**Solvents for chromatographic separations:** Different solvents are employed for chromatographic separation and purification procedures, including distilled water, acetonitrile (MeCN, highly flammable and toxic), methanol (MeOH, highly flammable and toxic), and formic acid (highly flammable, toxic, serious eye damage and corrosive). All solvents are analytical grade. Before use, these solvents must be filtered thought a PTFE membrane filter and sonicated to avoid air bubbles in the HPLC instrument.

**Instrumentation setup**

**HPLC-UV-MS:** The ESI-MS (Micromass ZQ2000 MS) detector with an electrospray interface is coupled with an HPLC system (Waters Alliance 2695) and a photodiode array detector (PDA, Waters 2996). Data acquisition and processing were performed using the Empower 2.0 software. The MS detector was optimized with the following conditions: capillary voltage: 3.00 kV, sample cone voltage: 30.0 V, source block temperature: 100 °C, desolvation temperature: 350 °C, operating in electrospray positive and negative modes, in the detection range *m*/*z* 200–800, with total ion count extracting acquisition. The cone and desolvation gas flows were 50 and 350 L.h^−1^, respectively, and were generated by a PEAK Scientific N110DR nitrogen source. The PDA scanned the samples at λ 200–800 nm. Samples were dissolved in MeOH at the concentration of 1 mg.mL^−1^. Analyses were performed using a reversed-phase Waters C_18_ X-Terra column (50 mm or 250 mm length) with different linear gradients of organic solvents (MeOH, MeCN, or MeOH/MeCN 1:1) in H_2_O with 0.1% formic acid during 12, 22, or 30 min, at a flow rate of 1 mL.min^−1^.

**UHPLC-HRMS/MS:** Analyses were performed on a Waters UPLC Xevo G2-XS Q-TOF equipment. UHPLC was performed at 0.5 mL.min^−1^ through a BEH C_18_ (2.1 × 100 mm; 1.7 μm, Waters) column at 40 °C. Samples were kept at 15 °C. Mobile phases were H_2_O (A) and MeCN (B), both acidified with 0.1% formic acid. The LC gradient was setup as follows: from 10% to 50% B for 6 min, from 50% B to 98% B for 3 min, from 98% to 10% B for 1 min for re-equilibration to initial conditions. ESI-HRMS/MS analyses were performed using data-dependent acquisition in positive mode with the following parameters: capillary voltage of 1.2 kV, cone voltage of 30 V, source temperature of 100 °C, desolvation temperature of 450 °C, cone gas (N_2_) flow of 50 L.h^−1^, desolvation gas (N_2_) flow of 750 L.h^−1^, scanning range *m*/*z* 100–1500 Da, scan time of 0.2 s, collision energy ramp of 6–9 V (low mass) and 60–80 V (high mass). For experiments with a focus on metabolomic diversity, the five most intense ions from each MS1 spectra were selected for MS/MS fragmentation. In the FFED experiments, the three most intense ions from each MS1 spectra were selected for MS/MS fragmentation. Internal calibration was accomplished with a solution of leucine enkephalin (200 pg.mL^−1^) infused by the lock-mass probe at a flow rate of 10 μL.min^−1^.

## Data Availability

The data presented in this study are available in previous publications [24,25,26,27,28,29].

## Supplementary Materials

The following supporting information can be downloaded at: https://www.mdpi.com/article/10.3390/mps6050077/s1. Table S1: Example of 1^st^-order effects calculated from global desirabilities (D) of fraction F2 obtained from each growth experiment (performed in triplicate) by *Penicillium* sp. DRF2; Table S2: Example of 2^st^-order effects calculated from global desirabilities (D) of fraction F2 obtained from each growth experiment (performed in triplicate) by *Penicillium* sp. DRF2; Figure S1: HPLC-UV analysis of fraction F2 produced by *Penicillium* sp. DRF2 under conditions of experiment #7.; Figure S2: HPLC-UV analysis of fraction F2 produced by *Penicillium* sp. DRF2 under conditions of experiment #11; Figure S3: HPLC-UV analysis of fraction F2 produced by *Penicillium* sp. DRF2 under conditions of experiment #17; Figure S4: HPLC-UV analysis of fraction F3 produced by *Penicillium* sp. DRF2 under conditions of experiment #11; Figure S5: HPLC-UV analysis of fraction F3 produced by *Penicillium* sp. DRF2 under conditions of experiment #17.

## Appendix A

Box A1 Castellani method: preservation on distilled water. A well-established and convenient preservation method of fungal strains is achieved by placing a piece of the fungi mycelium in sterile distilled water. The Castellani method is easy, inexpensive, and suitable for preservation of most species of fungi [43]. For that, inoculate the fungal strain into Petri dishes containing solid medium in which the strain was isolated, and incubate for 3 weeks at room temperature. After the growth, place a piece of the mycelium in Eppendorf tubes with autoclaved distilled water. Seal the tubes with Parafilm^®^, label, and store in a refrigerator at 4 °C.

Box A2 Quantification of a fungal spore suspension (spores.mL^−1^). The fungal spore suspension prepared as described in step 9 should be diluted to facilitate spore counting. A 1:10 dilution is suggested. An Eppendorf tube with 900 µL of distilled water is mixed with 100 µL of the spore suspension. If the counting is still difficult, a second dilution can be performed.
**Neubauer improved counting chamber:**
To count the spores into a Neubauer chamber, the guidelines described in the manufacturer’s manual should be followed to avoid double counting.

Box A3 Desirability function. Desirability is a mathematic function (equation) that identifies a common condition between several responses used in a factorial experimental design, herein exemplified between the mass quantity, number of chromatographic peaks, and chromatographic area recorded from a standard HPLC-UV-MS analysis. Each response will give a value of individual desirability (d_i_) that varies from 0 (a completely undesired response) to 1 (a completely desired response) [31].

$$d_{i}=\{\begin{array}{c} 0,\;if\;R<L\\ \frac{R-L}{T-L},\;if\;L\;\leq R\;\leq T\\ 1,\;if\;R>T \end{array}$$	R = average value of the response on triplicate experiments.
	L = the lowest response or the lowest desirable value.
	T = the highest response or the highest desirable value.

The global desirability (D) is the geometric average value of the individual desirabilities. For more details about the desirability function, see reference [31].

Box A4 Percentage calculus. The percentage (P_i_) calculus makes the analysis straightforward for interpretation. Using the equation below: each effect value (E_i_) is squared and divided by the sum of all the effects (first- and second-order) squared and multiplied by 100 [31].

$$P_{i}\left(\%\right)=\left(\frac{E_{i}^{2}}{\sum E_{i}^{2}}\right)\cdot 100$$

**Table A1:** Pre-selected variable levels used in the growth experiments to improve the production of secondary metabolites by fungi.

Variables	Levels	
	Low (−1)	High (+1)
1 Salts concentration in the culture medium, [salts]	10%	90%
2 Time of growth, t	7 days	35 days
3 Temperature of growth, T	15 °C	30 °C
4 Shaking or still, mode	0 rpm	100 rpm
5 Nutrients concentration in the culture medium, [nutrient]	20%	80%

**Table A2:** Fractional factorial experimental design (FFED) of five variables (2^(5−1)^ experiments).

GE	[ASW]	t	T	Mode	[M3]
1	−1	−1	−1	−1	+1
2	+1	−1	−1	−1	−1
3	−1	+1	−1	−1	−1
4	+1	+1	−1	−1	+1
5	−1	−1	+1	−1	−1
6	+1	−1	+1	−1	+1
7	−1	+1	+1	−1	+1
8	+1	+1	+1	−1	−1
9	−1	−1	−1	+1	−1
10	+1	−1	−1	+1	+1
11	−1	+1	−1	+1	+1
12	+1	+1	−1	+1	−1
13	−1	−1	+1	+1	+1
14	+1	−1	+1	+1	−1
15	−1	+1	+1	+1	−1
16	+1	+1	+1	+1	+1

**Table A3:** Conditions for each growth experiment as established by the FFED.

GE	[salts] (%)	t (days)	T (°C)	Mode (rpm)	[M3] (%)
1	10	7	15	0	80
2	80	7	15	0	20
3	10	35	15	0	20
4	80	35	15	0	80
5	10	7	30	0	20
6	80	7	30	0	80
7	10	35	30	0	80
8	80	35	30	0	20
9	10	7	15	100	20
10	80	7	15	100	80
11	10	35	15	100	80
12	80	35	15	100	20
13	10	7	30	100	80
14	80	7	30	100	20
15	10	35	30	100	20
16	80	35	30	100	80

**Table A4:** Addressing issues of the protocol for the marine-derived fungus *Roussoella* sp. DLM33 [27].

Step	Reason	Modification
10	The fungus did not sporulate.	The inoculum was prepared with four discs (3 mm) of the mycelium with 17 days of growth on culture plate.
11	Compound was not extracted with standard methodologies.	A mixture of EtOAc/MeOH/CH_2_Cl_2_ (6:3:1) with 1% formic acid was used to extract the mycelium + culture medium by sonicating (40 kHz) for 30 min.
14	Different time of growth.	The minimum level for the time variable was 14 days and the maximum 28 days.
18	The standard linear gradient of elution was not appropriate to efficiently detect compound **8**.	HPLC-UV-MS analysis was performed using a Waters C_18_ X-terra column (250 mm length) and a linear gradient of 40–100% MeOH in H_2_O for 30 min.
19	Different output.	The only response was the area under the chromatographic peak corresponding to the compound **8** under investigation.

**Table A5:** Addressing issues of the protocol for the marine-derived fungus *Biatriospora* sp. CBMAI 1333 [29].

Step	Reason	Modification
10	The fungus did not sporulate. Different culture media were used.	The inoculum was prepared with three discs (3 mm) of mycelium with 15 days of growth on a culture plate. The fungus was inoculated in 2% malt broth (M2), 3% malt broth (M3), glucose peptone yeast extract (GPY), potato dextrose broth (PDB), minimal medium (MM), Sabouraud dextrose (SBD), and SBD with yeast extract (SBD+YE) to select the best culture medium for the production of phomactins.
11	Shorter time of growth. Metabolites were not extracted with standard methods.	Time of growth was 15 days. Culture media were extracted as 12b and the remaining aqueous media phase was adsorbed in a mixture of XAD-2, -4, and -7 resins (1:1:1) for two days under shaking. The resin mixture was extracted with MeOH and 1:1 MeOH/acetone. Organic fractions and resin extracts were fractionated using cyanopropyl and C_18_-derivatized silica gel solid phase extraction cartridges.
12	MS/MS data was used to build molecular networks.	Clean extracts, fractions, and authentic standards of phomactins A, F, I, P, Q, R, S, and U were dissolved in MeOH and analyzed by UHPLC-HRMS/MS. Data were processed and imported into the GNPS platform to build molecular networks [44].
14	Different number of variables (n = 4).	Selected variables were growth temperature (15 °C or 30 °C), growth time (14 or 28 days), growth mode (shaken or still), and nutrient concentration (20% or 80% of nutrients of the original recipe for the MM medium in distilled H_2_O). A total of eight experiments were performed in three biological replicates each.
18	Different equipment used to run the analyses.	Samples were analyzed using UHPLC-HRMS/MS using data-dependent acquisition in positive mode and a gradient of MeCN in H_2_O, as described in Reagents Setup.
19	Different output.	Data of each individual UHPLC-HRMS/MS analysis were manually checked to obtain the results for each response variable ((i) number of chromatographic peaks corresponding to phomactins; (ii) total area of chromatographic peaks corresponding to phomactins; (iii) total weight of fractions containing phomactins).
