# Supplementary material for: Improvement of Targeted Fungi Secondary Metabolite Production Using a Systematic Experimental Design and Chemometrics Analysis
Source: Methods Protoc. 2023 Aug 29;6(5):77. doi: 10.3390/mps6050077 (PMC10514814; doi:10.3390/mps6050077)
Supplement: Supplementary file 1 [file mps-06-00077-s001.zip › mps-2521731-supplementary.pdf]

SUPPLEMENTARY INFORMATION

TABLE S1. Example of 1st-order effects calculated from global desirabilities (D) of fraction F2 obtained from each growth experiment (performed in triplicate) by *Penicillium* sp. DRF2.

TABLE S2. Example of 2st-order effects calculated from global desirabilities (D) of fraction F2 obtained from each growth experiment (performed in triplicate) by *Penicillium* sp. DRF2.

FIGURE S1. HPLC-UV analysis of fraction F2 produced by *Penicillium* sp. DRF2 under conditions of experiment #7.

FIGURE S2. HPLC-UV analysis of fraction F2 produced by *Penicillium* sp. DRF2 under conditions of experiment #11.

FIGURE S3. HPLC-UV analysis of fraction F2 produced by *Penicillium* sp. DRF2 under conditions of experiment #17.

FIGURE S4. HPLC-UV analysis of fraction F3 produced by *Penicillium* sp. DRF2 under conditions of experiment #11.

FIGURE S5. HPLC-UV analysis of fraction F3 produced by *Penicillium* sp. DRF2 under conditions of experiment #17.

TABLE S1. Example of 1st-order effects calculated from global desirabilities (D) of fraction F2 obtained from each growth experiment (performed in triplicate) by *Penicillium* sp. DRF2.

FFED							1st-order effect				
**#**	**1**	**2**	**3**	**4**	**5**	***D***	**1xD**	**2xD**	**3xD**	**4xD**	**5xD**
**1**	-1	-1	-1	-1	+1	0.26	-0.26	-0.26	-0.26	-0.26	0.26
**2**	+1	-1	-1	-1	-1	0.28	0.28	-0.28	-0.28	-0.28	-0.28
**3**	-1	+1	-1	-1	-1	0.39	-0.39	0.39	-0.39	-0.39	-0.39
**4**	+1	+1	-1	-1	+1	0.55	0.55	0.55	-0.55	-0.55	0.55
**5**	-1	-1	+1	-1	-1	0.06	-0.06	-0.06	0.06	-0.06	-0.06
**6**	+1	-1	+1	-1	+1	0.21	0.21	-0.21	0.21	-0.21	0.21
**7**	-1	+1	+1	-1	+1	0.66	-0.66	0.66	0.66	-0.66	0.66
**8**	+1	+1	+1	-1	-1	0.39	0.39	0.39	0.39	-0.39	-0.39

**9**	-1	-1	-1	+1	-1	0.58		-0.58	-0.58	-0.58	0.58	-0.58
**10**	+1	-1	-1	+1	+1	0.37		0.37	-0.37	-0.37	0.37	0.37
**11**	-1	+1	-1	+1	+1	0.61		-0.61	0.61	-0.61	0.61	0.61
**12**	+1	+1	-1	+1	-1	0.36		0.36	0.36	-0.36	0.36	-0.36
**13**	-1	-1	+1	+1	+1	0.13		-0.13	-0.13	0.13	0.13	0.13
**14**	+1	-1	+1	+1	-1	0.36		0.36	-0.36	0.36	0.36	-0.36
**15**	-1	+1	+1	+1	-1	0.36		-0.36	0.36	0.36	0.36	-0.36
**16**	+1	+1	+1	+1	+1	0.64		0.64	0.64	0.64	0.64	0.64
							**Σy^+**	**3.16**	**3.96**	**2.81**	**3.41**	**3.43**
							**Σy^-**	**-3.05**	**-2.25**	**-3.40**	**-2.80**	**-2.78**
							**$\Sigma y^+ + \Sigma y^-$**	**0.08**	**1.68**	**-0.58**	**0.61**	**0.65**
							**E_i (Σ/8)**	**0.01**	**0.21**	**-0.07**	**0.08**	**0.09**
							**P_i (%)**	**0.2**	**37.6**	**4.5**	**4.8**	**5.4**

TABLE S2. Example of 2nd-order effects calculated from global desirabilities (D) of fraction F2 obtained from each growth experiment (performed in triplicate) by *Penicillium* sp. DRF2.

			**2nd-order effect**									
**#**	***D***		**1x2xD**	**1x3xD**	**1x4xD**	**1x5xD**	**2x3xD**	**2x4xD**	**2x5xD**	**3x4xD**	**3x5xD**	**4xD**
**1**	0.26		0.26	0.26	0.26	-0.26	0.26	0.26	-0.26	0.26	-0.26	-0.26
**2**	0.28		-0.28	-0.28	-0.28	-0.28	0.28	0.28	0.28	0.28	0.28	0.28
**3**	0.39		-0.39	0.39	0.39	0.39	-0.39	-0.39	-0.39	0.39	0.39	0.39
**4**	0.55		0.55	-0.55	-0.55	0.55	-0.55	-0.55	0.55	0.55	-0.55	-0.55
**5**	0.06		0.06	-0.06	0.06	0.06	-0.06	0.06	0.06	-0.06	-0.06	0.06
**6**	0.21		-0.21	0.21	-0.21	0.21	-0.21	0.21	-0.21	-0.21	0.21	-0.21
**7**	0.66		-0.66	-0.66	0.66	-0.66	0.66	-0.66	0.66	-0.66	0.66	-0.66
**8**	0.39		0.39	0.39	-0.39	-0.39	0.39	-0.39	-0.39	-0.39	-0.39	0.39
**9**	0.58		0.58	0.58	-0.58	0.58	0.58	-0.58	0.58	-0.58	0.58	-0.58
**10**	0.37		-0.37	-0.37	0.37	0.37	0.37	-0.37	-0.37	-0.37	-0.37	0.37
**11**	0.61		-0.61	0.61	-0.61	-0.61	-0.61	0.61	0.61	-0.61	-0.61	0.61
**12**	0.36		0.36	-0.36	0.36	-0.36	-0.36	0.36	-0.36	-0.36	0.36	-0.36
**13**	0.13		0.13	-0.13	-0.13	-0.13	-0.13	-0.13	-0.13	0.13	0.13	0.13
**14**	0.36		-0.36	0.36	0.36	-0.36	-0.36	-0.36	0.36	0.36	-0.36	-0.36
**15**	0.36		-0.36	-0.36	-0.36	0.36	0.36	0.36	-0.36	0.36	-0.36	-0.36
**16**	0.64		0.64	0.64	0.64	0.64	0.64	0.64	0.64	0.64	0.64	0.64
		**Σy^+**	**2.97**	**3.44**	**3.10**	**3.16**	**3.54**	**2.78**	**3.74**	**2.97**	**3.25**	**2.87**
		**Σy^-**	**-3.24**	**-2.77**	**-3.11**	**-3.05**	**-2.67**	**-3.43**	**-2.47**	**-3.24**	**-2.96**	**-3.34**
		**$\Sigma y^+ + \Sigma y^-$**	**-0.27**	**0.67**	**-0.01**	**0.11**	**0.87**	**-0.65**	**1.27**	**-0.27**	**0.29**	**-0.47**
		**E_i (Σ/8)**	**-0.03**	**0.08**	**0.00**	**0.01**	**0.11**	**-0.08**	**0.16**	**-0.03**	**0.03**	**-0.06**
		**P_i (%)**	**0.9**	**5.8**	**0.0**	**0.2**	**9.7**	**5.4**	**20.7**	**0.9**	**1.1**	**2.8**

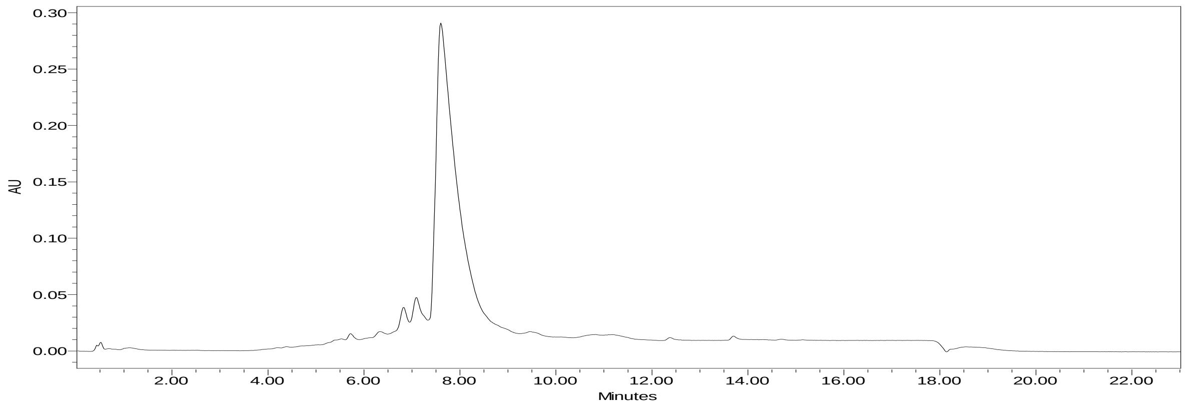

FIGURE S1. HPLC-UV analysis of fraction F2 produced by *Penicillium* sp. DRF2 under conditions of experiment #7.

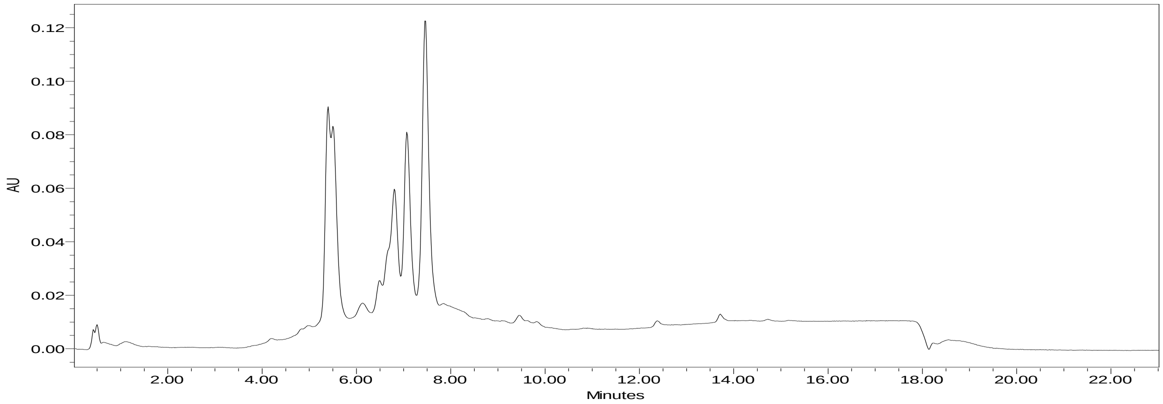

FIGURE S2. HPLC-UV analysis of fraction F2 produced by *Penicillium* sp. DRF2 under conditions of experiment #11.

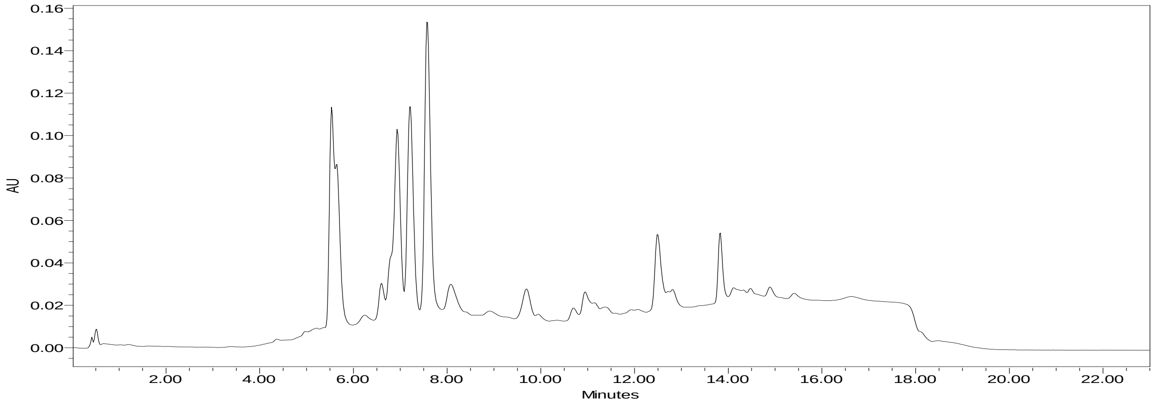

FIGURE S3. HPLC-UV analysis of fraction F2 produced by *Penicillium* sp. DRF2 under conditions of experiment #17.

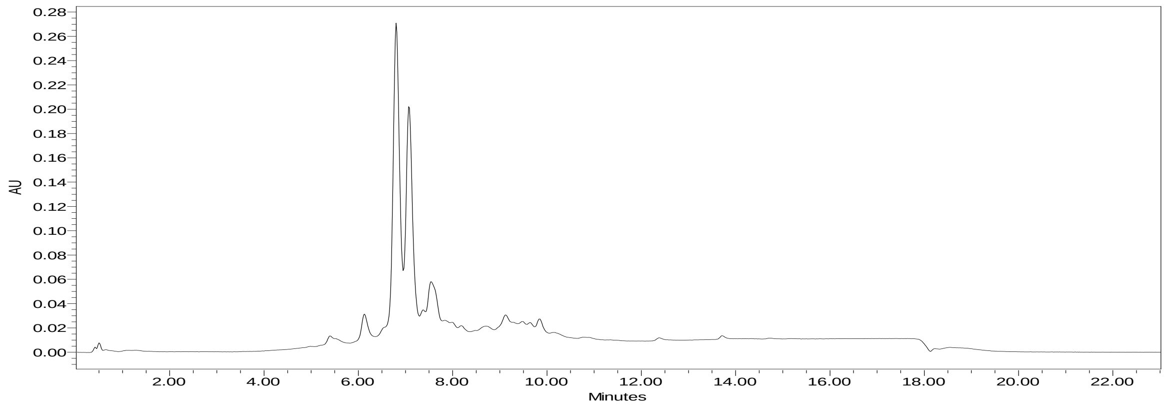

FIGURE S4. HPLC-UV analysis of fraction F3 produced by *Penicillium* sp. DRF2 under conditions of experiment #11.

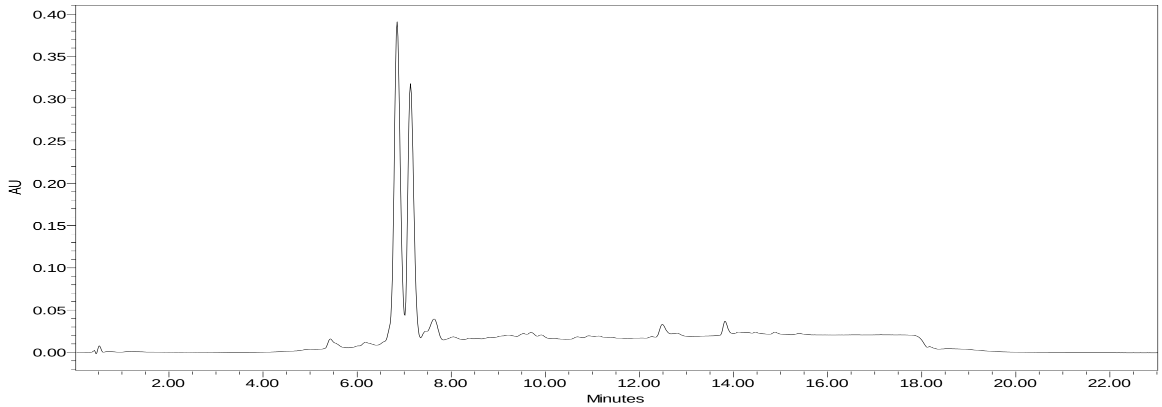

FIGURE S5. HPLC-UV analysis of fraction F3 produced by *Penicillium* sp. DRF2 under conditions of experiment #17.
